# Impaired Wnt signaling in dopamine containing neurons is associated with pathogenesis in a rotenone triggered *Drosophila* Parkinson’s disease model

**DOI:** 10.1038/s41598-018-20836-w

**Published:** 2018-02-05

**Authors:** Flora Stephano, Stella Nolte, Julia Hoffmann, Samar El-Kholy, Jakob von Frieling, Iris Bruchhaus, Christine Fink, Thomas Roeder

**Affiliations:** 1 Kiel University, Zoological Institute, Department Molecular Physiology, Kiel, Germany; 2 Bernhard-Nocht Institute for Tropical Medicine, Hamburg, Germany; 3 University of Dar es Salaam, Department of Zoology and Wildlife Conservation, Dar es Salaam, Tanzania; 4 Tanta University, Faculty of Science, Department of Zoology, Tanta, Egypt; 5 Airway Research Center North of the German Center for Lung Research (DZL), Grosshansdorf, Germany; 6 Present Address: Aarhus University, Dandrite, Department Molecular Biology and Genetics, Aarhus, Denmark; 7 Present Address: Amedon AG, Lübeck, Germany

## Abstract

Parkinson’s disease, which is the one of the most common neurodegenerative movement disorder, is characterized by a progressive loss of dopamine containing neurons. The mechanisms underlying disease initiation and development are not well understood and causative therapies are currently not available. To elucidate the molecular processes during early stages of Parkinson’s disease, we utilized a *Drosophila* model. To induce Parkinson’s disease-like phenotypes, we treated flies with the pesticide rotenone and isolated dopamine producing neurons of animals that were at an early disease stage. Transcriptomic analyses revealed that gene ontologies associated with regulation of cell death and neuronal functions were significantly enriched. Moreover, the activities of the MAPK/EGFR- and TGF-β signaling pathways were enhanced, while the Wnt pathway was dampened. In order to evaluate the role of Wnt signaling for survival of dopaminergic neurons in the disease model, we rescued the reduced Wnt signaling activity by ectopic overexpression of *armadillo*/β-catenin. This intervention rescued the rotenone induced movement impairments in the *Drosophila* model. Taken together, this initial study showed a highly relevant role of Wnt signaling for dopamine producing neurons during pathogenesis in Parkinson’s disease and it implies that interfering with this pathway might by a suitable therapeutic option for the future.

## Introduction

Parkinson’s disease (PD) is the second most prevalent neurodegenerative disorder with an incidence of about 1% in people older than 65 years^1,2^. Patients suffering from PD show a selective and progressive loss of dopaminergic (DA) neurons and the presence of Lewy bodies in surviving neurons in the *Substantia nigra*^3^. The most important symptoms are motor system disturbances such as bradykinesia, resting tremor, rigidity and postural instability. These symptoms appear when degeneration of DA neurons is at an advanced stage. Currently, no causative treatment strategy is available for this complex disease, where both, genetic susceptibility and environmental factors contribute to induce pathogenesis. Although epidemiological and genetic studies yielded important insights into causes underlying pathogenesis, the precise pathologic molecular mechanisms remain unclear^1,4–6^.

The identification of genes causatively related to familiar forms of the disease raised the hope to identify common cellular signatures that underlie pathogenesis^7,8^. However, a detailed understanding of the first steps that trigger Parkinson’s disease (PD) is currently almost completely missing. The major strategy followed by most researchers in the field aims to understand the effects caused by mutations in genes linked to PD including *Parkin*, *Pink*, *SCNA* or *LRRK2*. Alternatively, a detailed understanding of common signaling pathways that are deregulated during pathogenesis might pave the way for a better understanding of disease development. A very promising candidate that appears to be deregulated during disease development is the canonical Wnt pathway. Evidences that link deregulated Wnt signaling with a number of neurological disorders have already been highlighted^9,10^. In Alzheimer’s disease (AD), an impairment of canonical Wnt signaling has been tightly associated with early disease phases. There, amongst other pathway components, Dickkopf-1 (Dkk1) levels are enhanced, which effectively inhibits Wnt signaling^11^. Other risk factors for AD such as specific ApoE4- or LRP6 variants are also known to reduce neuronal Wnt signaling^12^. Although, an association between early stages of the disease and impaired Wnt signaling has also been shown in PD^8,13,14^, it is not generally accepted that deregulated Wnt signaling is indeed a driving force underlying PD development. To study these early molecular events in DA neurons that finally lead to PD, animal models have been the foremost tools. Rodent models of PD, especially murine ones have been extremely successful, despite the fact that they do not recapitulate all facets of the disease^15^. Thus, alternative animal models, including those utilizing invertebrates are highly relevant. Very simple and genetically tractable model became very attractive with a series of different *Drosophila* model being the most successful ones^16^. Targeted overexpression of human alpha-synuclein induces symptoms typically seen in human PD patients similarly as other PD susceptibility genes did^17–19^. Moreover, simple, pharmacologically induced PD model that utilize either the pesticide rotenone or paraquat to trigger DA neuronal impairment were introduced in flies. They showed a very impressive phenotype, a substantially reduced and quantifiable climbing ability^20^. The different *Drosophila* models of PD have been very informative in order to identify mechanisms underlying disease development and potential interventions that delay or prevent it. Very impressive were the observations that either coffee and cigarette smoke induce Nrf2-mediated protection in these models of PD^21,22^.

In the current study, we focused on understanding molecular responses in DA neurons during the early phase of PD, where impairments of the motor abilities are still not detectable, in order to identify potential targets for therapeutic intervention. We used rotenone to induce Parkinsonism in the fly and combined this with a cDNA microarray analysis of DA neurons that were isolated using a magnetic bead based approach. Our data provide evidences that the outcomes of various highly relevant molecular signaling pathways are modified in DA neurons that are in an early stage of PD. Amongst these affected systems are the Wnt-, MAPK/EGFR-, TGF-β-, and TOR-signaling pathways, which are known to be important for cell survival and/or cell death in the CNS.

## Results

### Rotenone induced changes in locomotor behavior in an administration time dependent manner

**Figure 1:**
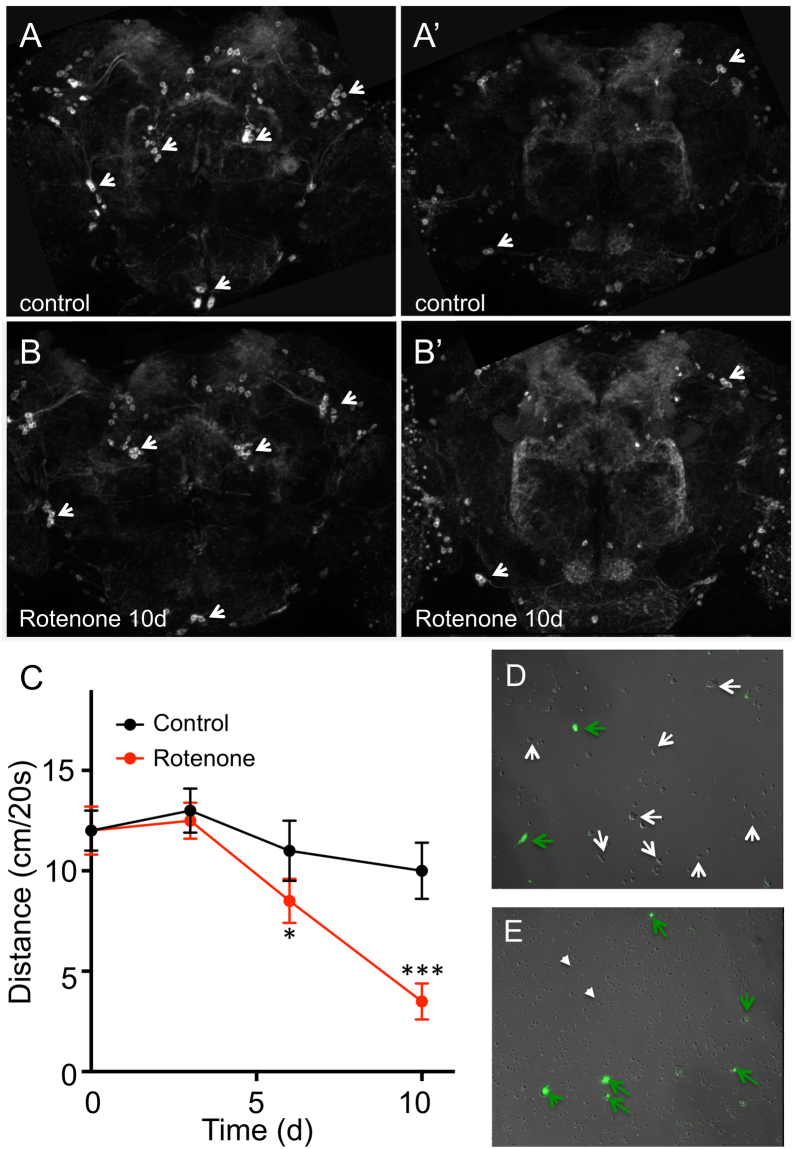
Isolation of dopamine producing cells following rotenone treatment. Brains of adult animals of the genotype TH-GAL4 > 20xUAS-mCD8::GFP from control animals (**A,A′**) as well as from animals treated for 10 d with rotenone (**B,B′**) were analyzed using anti GFP immunohistochemistry. GFP-positive, dopamine containing neurons are shown (arrows) in two different Z-areas (**A,A**′,**B,B′**). (**C**) Exposure of rotenone for different periods induced climbing disability in a time dependent manner. The distance reached by animals climbing a vertical plane within 20 s is given. Shown are mean values of three independent experiments, ±SEM, n = 20 individuals for each group (**P < 0.01, ***p < 0.005). (**D**,**E**) Magnetic bead assisted isolation of dopamine containing cells using beads coated with anti-mCD8 antibodies. (**D**) Cell suspension of decollated neurons containing GFP-positive (green arrows) as well as non-labeled neurons (white arrows). (**E**) GFP-positive cells (green arrows) and magnetic beads (white arrow heads) after bead-assisted isolation devoid of non-labeled cells.

This study aims to elucidate the molecular changes occurring in DA neurons during early phases of disease development. PD like symptoms were induced by rotenone application. Previous studies have shown that detectable motor impairments and DA neuron loss start after chronic exposure with rotenone for several days and that this symptomatic phase starts after the majority of DA neurons were already severely damaged^20,23^. This severe motor impairment is known to be not associated with a concurrent demise of dopaminergic cells^24^. In order to monitor the onset and development of the PD-associated pathologies, we used our experimental animals (F1 of *TH-GAL4* X *20xUAS-mCD8::GFP*) and treated them with rotenone (Fig. 1). Brains of non-treated animals (Fig. 1A,A′) and of rotenone treated (0.5 mM for 10 d; Fig. 1B,B′) were analyzed. Shown are two regions per treatment type (control, A,A′; rotenone for 10d, B,B′) of representative brains of the above mentioned genotype (*TH-GAL4 X 20xUAS-mCD8::GFP*) labeled with anti-GFP antibodies. The *TH-Gal4* driver line labels about 50% of all dopaminergic cells in the fly’s brain^25^. The number and location of neurons remained mostly unchanged, which was in line with previous observations^24^. In non-treated control animals, we observed a mean of 52.13 (±2.1, S.E.M) GFP-positive cells per hemisphere, whereas we identified 49.5 (±5.3, S.E.M.) cells per hemisphere in rotenone- treated animals. This difference was not statistically significant (p = 0.92, N = 8). In contrast, treatment with the sublethal dose of 0.5 mM rotenone had a significant effect on the motor abilities of the treated flies. We quantified their ability to climb a vertical plane (Fig. 1C). There was no obvious locomotor deficit observed in flies exposed to rotenone for up to 3 days. From day 6 onwards, flies showed statistically reduced locomotor ability in comparison to untreated flies, meaning that their mean climbing distance was reduced substantially (Fig. 1C). Thus, we choose flies treated for three days with rotenone for all subsequent transcriptomic analyses. At this time point, we can assume that the first signs of pathological alterations had been induced, but still before the onset of the phenotypical hallmark, the locomotor impairment, of this disease model. Consequently, we performed whole transcriptome analysis with RNA isolated specifically from DA neurons after this time of treatment to ensure that those effects can be identified that occur prior to development of the disease associated phenotype. In parallel, parts of the experimental populations were used for phenotypical analyses of the rotenone induced Parkinsonism over time, which always showed the kinetics as outlined in Fig. 1C. In order to isolate the mCD8::GFP tagged neurons, they were decollated first (Fig. 1D). The figure shows few GFP-positive cells (TH-Gal4 positive) and numerous other, non-labeled neurons. Using an isolation protocol based and anti-mCD8 coupled magnetic beads, the mCD8-GFP-tagged cells were isolated and all non-labeled cells depleted from the preparation. The high degree of enrichment of mCD8-GFP-positive cells is shown in Fig. 1E, where some GFP-positive cells (green arrows), but no non-labeled cells are detectable.

### Rotenone induced gene expression changes in DA neurons in an early phase of PD

**Figure 2:**
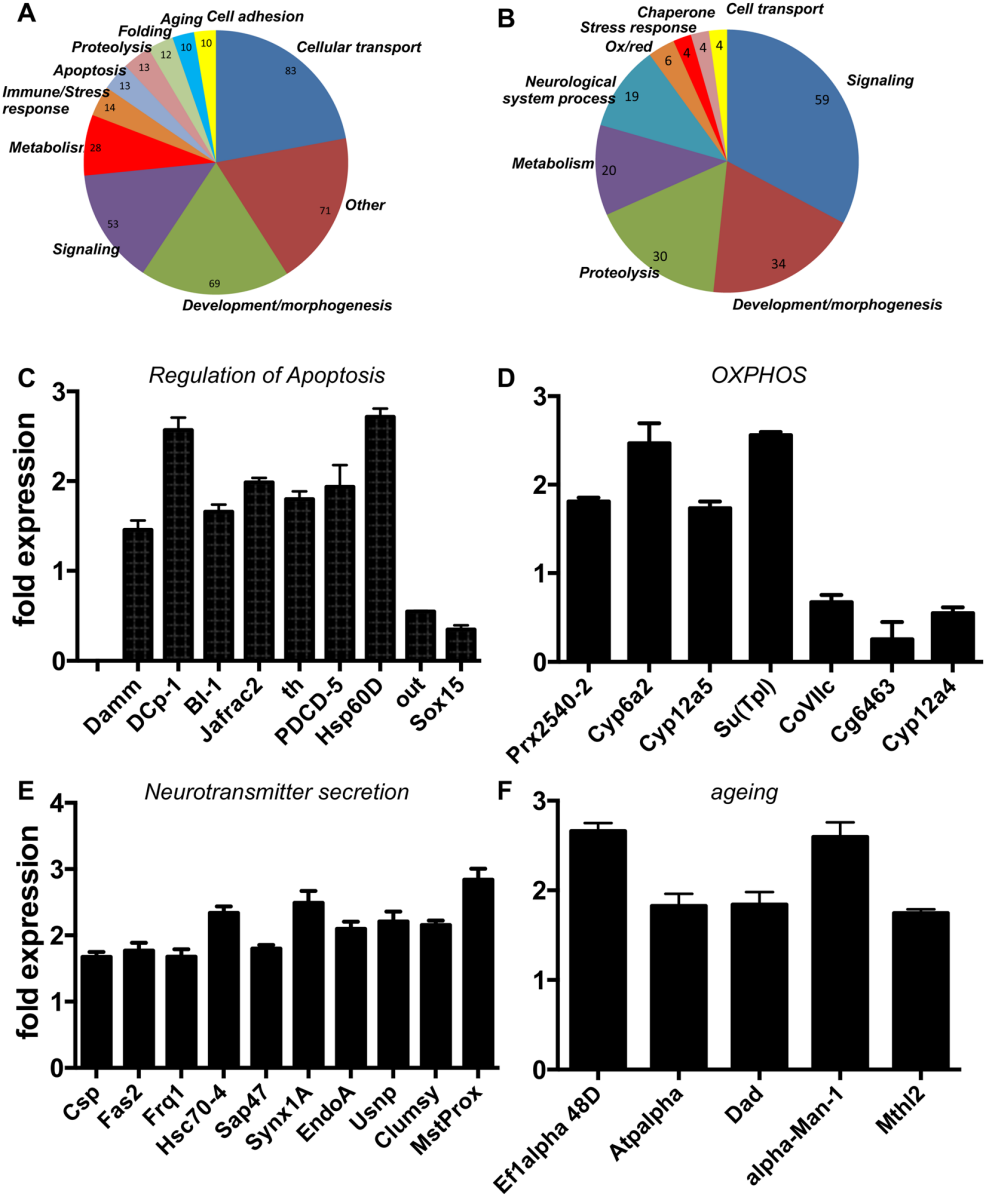
Early transcriptional response to rotenone treatment of dopamine containing cells. GO analyses of genes that were upregulated (**A**) or downregulated (**B**) in dopamine containing cells in response to rotenone treatment. Clustering was performed with the DAVID program package^68^. Functional categories overrepresented in the lists of genes differentially expressed in rotenone treated flies identified by DAVID (Table 1, p < 0.05). (**C**) Fold changes for genes associated with the GO term regulation of apoptosis (**D**) Oxidative phosphorylation (**E**) neurotransmitter secretion (**F**) ageing. Error bars represent SEM. Damm: Death associated molecule related to Mch2, Dcp-1: Death caspase 1, BI-1: Bax Inhibitor 1, Jafrac2: Thioredoxin peroxidase 2, th: thread, PDCD-5: Programmed cell death 5, Hsp60D, out: Outsiders, sox15: Sox box protein 15, Prx2540-2: Peroxiredoxin 2540-2, Cyp6a2, Cyp12a5, Cyp12a4: Cytochrome p450-6a2, 12a4, 12a5, respectively, Su(Tpl): CoVIIc: Cytochrome c oxidase subunit VIIc, CG6463, Csp: Cysteine string protein, Fas2: Fasciclin2, Frq1: Frequenin 1, Hsc70-4: Heat shock protein cognate 4, Sap47: Synapse associated protein 47kD, Syx1A: Syntaxin 1A, EndoA: Endophilin A, Usnp: Ubisnap, clumsy, and MstProx, Ef1alpha 48D: Elongation factor 1 alpha 48D, Atpalpha: Sodium pump alpha subunit, Dad: daughter against dpp, alpha-Man-1: alpha-Mannosidase class 1a and Mthl2: methuselah-like 2.

Although flies treated with 0.5 mM rotenone for three days appear to coordinate their movement normally (Fig. 1B), a number of genes show differential expression in DA neurons obtained from rotenone treated animals compared with those isolated from sham-treated ones. Expression of 765 genes was up-regulated (>1.5 fold) and that of 357 genes was down-regulated (<0.67 fold) if compared with the signals obtained from DA neurons isolated from sham-treated animals. To further filter and identify relevant regulated genes in the two lists, we used different Gene Ontology (GO) analysis programs such as the DAVID^26^ and the GOrilla program packages^27^. Different GO terms were found to be enriched in either the cohorts of up- or down-regulated genes or of both groups together. The GOrilla analysis revealed a significant enrichment of the GO terms regulation of cell death and negative regulation of cell death (p values of 8.8 10^−4^ and 5.3 10^−5^, respectively). Moreover, terms such as negative regulation of cell cycle were also enriched. Applying the DAVID program package to the data revealed additional GO terms as being overrepresented. Those genes with functions related to cellular transport were changed with the highest frequency (83 genes), followed by various others (Fig. 2A). Other important categories are related to stress response (14 genes), regulation of apoptosis (13 genes), protein folding (12 genes) and ageing (10 genes) (Fig. 2A). These results might be interpreted as a defense response towards a mild toxic offence given by rotenone. Likewise, a list of down-regulated genes was generated and clustered into 9 relevant functional categories (Fig. 2B).

Collectively, GO terms that were highly enriched in the combined cohorts of up- and down-regulated genes included regulation of apoptosis (Fig. 2C), oxidative phosphorylation (OXPHOS, Fig. 2D), neurotransmitter secretion (Fig. 2E) or ageing (Fig. 2F). Additional relevant clusters with high enrichment score and p < 0.05 and KEGG pathways filtered by DAVID are listed in Table 1 and Fig. S1, respectively. To validate the differential expression obtained using the microarray data, we performed qRT-PCR experiments with 3 informative genes, namely down-regulation of *dat* (*Dopamine N-acetyltransferase*) as well as the enhanced expression of *calmodulin* and of *lethal2NC136* (Fig. S2).

**Table 1:** Enriched functional categories for genes differentially regulated by rotenone.

Database identifier	Functional category (Enriched GO terms)	Number of changed genes	p-value < 0.05
**Upregulated genes**			
GO:0032535	Regulation of cell size/Neurogenesis	13	0.022
GO:0042981	Apoptosis	10	0.030
GO:0007568	Ageing	10	0.030
KEGG_PATHWAY	MAPK signaling pathway	6	
KEGG_PATHWAY	mTOR signaling pathway	4	
KEGG_PATHWAY	TGF beta signaling pathway	5	
SM00034	CLECT C-type	6	0.040
GO:0007269	Cell-cell signaling/neurotransmitter secretion	15	0.040
GO:0000022	Motor activity	11	0.003
GO:0046146	Tetrahydrobiopterin metabolic process	4	0.012
**Downregulated genes**			
GO:0050877	Neurological system processes	20	0.014
KEGG_PATHWAY	Wnt signaling pathway	6	
GO:0010564	Regulation of cell cycle process	6	0.024
GO:0009310	Amine catabolic process	4	0.040

### Pathway enrichment analysis

**Figure 3:**
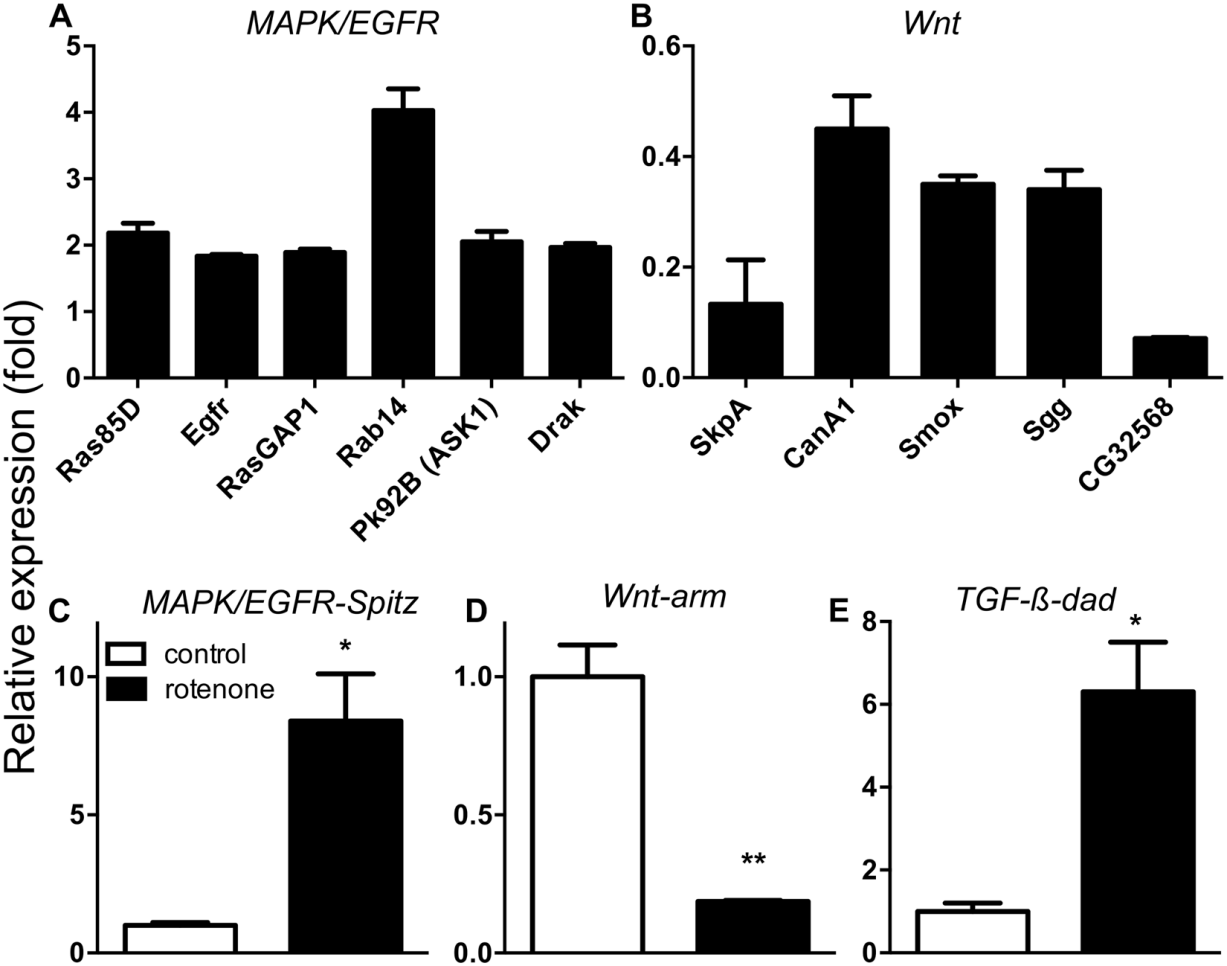
Early modifications of signaling pathways in dopamine containing neurons in a rotenone induced PD model. (**A**) Shows up-regulated genes associated with MAPK/EGFR signaling pathway derived from the microarray data, and down-regulated genes associated with Wnt signaling pathway (**B**). qRT-PCR analysis of canonical target genes of the corresponding pathways (**C**–**E**). Spitz, a canonical target gene of the EGFR-pathway (**C**), Armadillo (arm) a central mediator of the wnt-pathway (**D**) and dad, a canonical TGF-β-pathway response gene (**E**). Ras85D: Ras Oncogene at 85D, Egfr: Epidermal growth factor receptor, RasGAP1: Ras GTPase activating protein 1, Rab14, Pk92B or DASK1: Protein kinase at 92B, drak: Death associated protein kinase related, skpA:skpA, CanA1: Calcineurin A1, smox: Smad on X, sgg: Shaggy: CycD: Cyclin D.

Since pathway analysis is anticipated to give more instructive data than single gene analysis^28^, further analyses focused on those pathways, whose activity was apparently deregulated. KEGG analysis revealed four pathways to be significantly altered, namely; the mitogen-activated protein kinases (MAPK/EGFR), the transforming growth factor beta (TGF-β), the target of rapamycin (Tor) and the Wnt signaling pathway. While activity of the first three pathways appear to be increased, that of the Wnt pathway appears to be reduced (Fig. 3, Table 1). Genes tightly associated with the MAPK/EGFR signaling are shown in Fig. 3A, while those associated with the Wnt pathway are shown in Fig. 3B. Expression levels of genes overrepresented in TGF-β and Tor signaling are shown in Table S1. In order to evaluate if the activity of the corresponding pathways were indeed induced or impaired, we analyzed either canonical pathway genes of these pathways or essential pathway constituents by qRT-PCR. For the MAPK/EGFR-pathway, expression of the canonical pathway gene *spitz*^29^ was upregulated several fold (*p < 0.05; Fig. 3C). For Wnt signaling we analyzed *armadillo*, a central regulatory component of the Wnt signaling pathway (the β-catenin homolog). The relative expression level of *armadillo* in rotenone treated samples was significantly reduced (**p < 0.01; Fig. 3D). For the TGF-β signaling pathway, the expression level of its canonical target gene, *Daughter against dpp* (*Dad*) was analysed. Its expression level was significantly increased in rotenone treated samples (*p < 0.05; Fig. 3E), indicative for an increased pathway activity.

**Figure 4:**
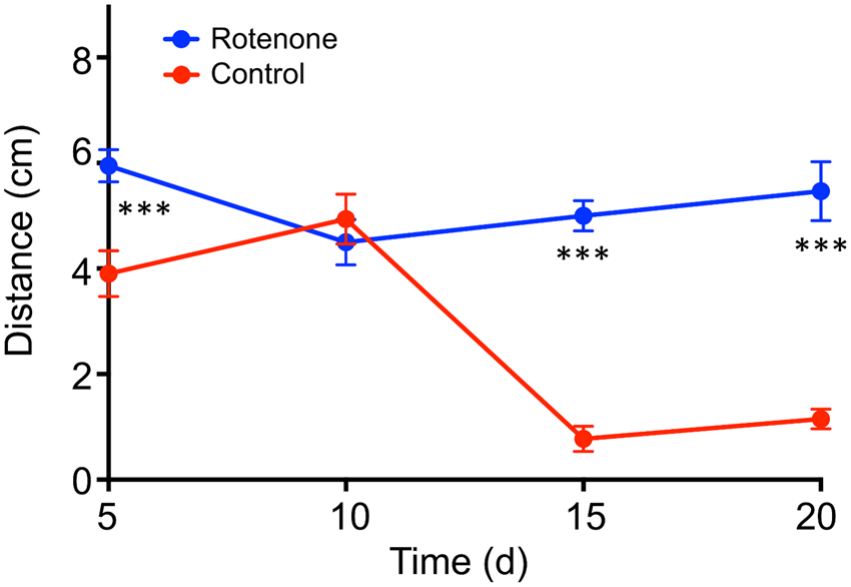
Effect of *armadillo* overexpression in dopamine-containing cells in the rotenone induced PD model. *Armadillo* was specifically overexpressed by using TH-Gal4/UAS-arm animals. All animals were subjected to rotenone treatment and the climbing abilities of the experimental (blue) as well as of the control animals (red, background line for both insertions *w*^*1118*^, control = *w*^*1118*^) were analyzed. The distance reached by animals climbing a vertical plane within 10 s is given (N = 6 independent experiments, mean ± SEM, ***p < 0.001).

In order to evaluate the relevance of modulating the candidate signaling pathways, we employed the Gal4/UAS-system. Using the *TH-Gal4* driver line, this manipulation was restricted to DA containing cells only. We focussed on overexpression of *armadillo* (using *UAS-arm*) as the most important signalling pathway impaired by rotenone treatment. While the climbing performance index (relative proportion of animals that were able to cross a 2 cm threshold within 10 s) was identical between controls and those overexpressing armadillo were identical at the beginning of rotenone treatment, the decline in performance from day 10 onwards was dramatic while this was not the case for animals with ectopically increased armadillo expression in dopamine-producing cells only (Fig. 4). These differences were statistically highly significant.

## Discussion

The major aim of the current study was to provide new information about the molecular signatures associated with early phases of an induced Parkinson’s disease like phenotype that develop specifically in dopamine containing neurons of the brain. In order to reach this ambitious goal, focusing exclusively on these dopamine producing cells being in an early stage was mandatory. *Drosophila* is ideally suited for this purpose as PD-like phenotypes can be induced easily, their progression can be quantified non-invasively and the dopamine-producing cells can be labeled specifically. Analyzing the early stage is of prime importance, because, at least in humans, clinically noticeable phenotypes occur after an estimated 70% of susceptible DA neurons in the *Substantia nigra* have already been destroyed^30^. Although *Drosophila* models of Parkinson’s disease recapitulate most aspects of the disease, this appears to be based on massive functional impairments of dopaminergic cells rather than on their death^24^. Thus, we analyzed in the current disease model a very early stage of cell pathology that later develops into the disease-associated functional impairments. We used a toxin-induced model of Parkinson’s disease to enhance the freedom of operation for genetic manipulation. Although we decided to utilize rotenone treatment, rotennone and paraquat models are equally well-suited for the induction of Parkinson’s disease symptoms in the fly^24,31^. The gene expression analysis carried out in this study is, to our knowledge, the first for rotenone-induced Parkinsonism in *Drosophila* that was focused on DA neurons. Prior studies of gene expression in *Drosophila* models of neurodegenerative diseases have been limited to studies of homogenates of brain tissue^32,33^. In contrast, a comparable approach has been performed in mammals, namely in rats. Rotenone treatment followed by laser-dissection and transcriptome analyses of dopaminergic neurons revealed complex sets of regulated genes^34^. A closer comparison of the sets of genes that are regulated in dopamine-containing neurons of rats and flies revealed some surprising commonalities in their response characteristics to rotenone treatment. Regulated Gene Ontologies comprise those associated with cell death and cell cycle as well as those directly associated with general neuronal activities such as neurotransmitter release, which implies that the reaction types of rat and fly dopamine-containing neurons are surprisingly similar. In human brain tissues, the laser capture microdissection (LCM) technique has recently been employed for capturing only DA neurons for transcriptional profiling studies^35,36^. The complex profile of genes with differentially regulated expression in response to rotenone treatment comprises some highly enriched pathways and gene ontologies. Presumably most relevant was the regulation of signaling pathways in these cells that are known to be highly relevant for cell survival and cell death, such as the MAPK/EGFR, TGF-β, Tor and Wnt pathways.

Of special interest in this study was the observed down-regulation of Wnt signaling in response to mild rotenone treatment in young adult flies. Armadillo, the *Drosophila* β-catenin acts as a transcription factor inducing expression of Wnt target genes. Wnt signaling is known throughout the animal kingdom to be involved in controlling diverse cellular processes including tissue differentiation, neuronal survival, synaptogenesis and plasticity, as well as neurogenesis and neuroprotection^9,37,38^. Moreover, deregulated Wnt signaling is believed to be involved in various neuropathologies including Alzheimer’s disease, Schizophrenia and PD^8,10^. Canonical Wnt/β-catenin signaling appears to be highly important for controlling DA neuronal fate decision^39^. Remarkably, both GSK3-β inhibition and β-catenin stabilization increased commitment of neural precursors to develop into DA neurons^40^. Importantly, current studies imply that PD pathophysiology is associated with dysregulation of Wnt signaling^8,9,14,41^. Cantuti-Castelverti and colleagues reported a down-regulation of β-catenin levels in DA neurons of the *Substantia nigra* in PD patients^36^. In addition, proteins encoded by PARK genes, which were also shown to be involved in hereditary forms of PD, can modify Wnt pathway activity. LRRK2 (leucine-rich repeat kinase 2), which is associated with familial PD^42^ was shown to be connected to Wnt signaling^8,13^ and Parkin, an E3 ubiquitin ligase, regulates β-catenin protein levels *in vivo*^14^. Furthermore, gene expression profiling in progressively MPTP-lesioned macaques indicated down-regulation of β-catenin and dysregulation of key components of Wnt signaling^28^. Our results are consistent with these discussed findings, suggesting a general role of Wnt signaling in DA neuron welfare and PD development upon its impairment. Apparently, a certain level of Wnt signaling is necessary to guarantee survival of dopamine-containing neurons, especially in times of stress. Our rescue experiments support the view that downregulation of Wnt signaling is a key event in the neuropathology of PD and that amending this impairment improves the functionality and presumably also the survival of DA neurons at risk. Thus, interfering with Wnt signaling in DA containing neurons may represent a very promising and novel therapeutic strategy.

Very similar as Wnt signaling, MAPK signaling is evolutionarily well conserved. *Drosophila melanogaster* expresses all three subgroups of MAPKs: Rl (Rolled; ERK homolog), dJNK/Basket (*Drosophila* homolog of JNK), and dp38a and dp38b (*Drosophila* homologs of p38)^43,44^. Impairments of MAPK signaling pathways have been associated with many diseases including Alzheimer’s, PD and diverse types of cancer^45^. Strikingly, even at this early stage of disease development, expression of four genes associated with the MAPK signaling pathway was approximately 2-fold increased in rotenone treated flies. Moreover, expression of the *Egfr*, which is the central receptor in this signaling pathway, was also increased. Mice lacking the EGFR develop neurodegenerative diseases and die early^46^. In culture models, EGF stimulated neurite outgrowth, increased dopamine uptake and enhanced long-term survival in cultured dopaminergic neurons. Ectopic activation of ERK1/2 in rotenone rat models of PD, on the other hand, protected dopamine neurons from cell death^47^. In *Drosophila*, proper functioning of EGFR signaling has been shown to be essential for learning and memory^48^. The increased expression of *EGFR* and *Ras* protein coding genes in rotenone treated *Drosophila* suggests that these neurons launch defensive mechanisms due to stress given by rotenone. These findings support the view that EGFR signaling promotes cell survival also in the vulnerable DA neurons.

The third highly relevant signaling system, the TGF-β pathway controls a surplus of cellular processes in both developing and adult organisms^49,50^. When components of the TGF-β pathway are disrupted, several human diseases, including neurodegeneration and cancer arise^50–52^. There is increasing support for a role of TGF-β signaling in neuronal maintenance function and degeneration^52–54^.

As most of the other pathways mentioned above, the target of rapamycin (TOR) signaling pathway is evolutionary conserved. It regulates cell proliferation, cell motility, cell survival, protein synthesis and transcription^55,56^. Nucleolar disruption and associated oxidative stress were demonstrated to suppress mTOR activity in DA neurons, thereby providing the basis for neuronal degeneration and the development of parkinsonism^57^. However, major key players of the pathway (*TOR*, *Akt* and *elF4B*) appeared to be up-regulated in flies treated with rotenone in the early phase, which is indicative for induction of survival mechanisms.

Collectively, this study shows that the expression of several genes involved in the MAPK/EGFR, TGF-β and TOR signaling pathways were increased, presumably in order to launch a protective cellular program. The observed down-regulation of Wnt signaling on the other hand may reflect early signs of neurodegeneration. Thus, increasing expression of either of the pathways mentioned above may increase survival of dopamine-containing neurons during disease progression, a hypothesis that is supported by the first experiments performed in this study focusing on enhancing Wnt signaling.

## Methods

### Fly strains and husbandry

Fly stocks were raised on standard cornmeal-agar medium at 25 °C under 12 h/12 h on/off light cycle. The following flies were used in the experiments described below: *TH-Gal4* males (Bloomington *Drosophila* Stock Center, USA) were crossed to virgin females of either *20xUAS-IVSmCD8::GF*P or *UAS-arm* (Bloomington *Drosophila* Stock Center, USA) to generate flies overexpressing *GFP* or *armadillo* in dopaminergic neurons, respectively.

### Toxin administration

Rotenone (Sigma Aldrich, Deisenhofen, Germany) was administered orally according to a previously described procedure^23^ with minor modifications. Briefly, 3-day- old flies from the crossing mentioned above were exposed to 0.5 mM rotenone in 10% glucose on blotting paper at 25 °C. A volume of 300 µl rotenone/glucose solution was added every 48 h to avoid desiccation. For the control group the same volume of 10% glucose only was added.

In order to evaluate the relevance of selected signaling pathways for survival of dopamine containing neurons in the presence of rotenone, the Gal4/UAS system was employed utilizing the TH-Gal4 driver and effector lines used to ectopically overexpress relevant genes.

### Immunohistochemistry

Immunohistochemistry was performed as previously described^58^. Brains were dissected manually in *Drosophila* Ringer’s solution and immediately fixed in 4% paraformaldehyde in PBS for 30 min at room temperature. Subsequently, the samples were washed with PBST (0.3% Triton X-100 in PBS) and blocked in blocking-buffer (10% goat serum in PBST) for 30 min at room temperature, followed by incubation with the primary antibody (1:300 rabbit anti-GFP, Sigma-Aldrich, Taufkirchen, Germany) overnight at 4 °C with subsequent application of the secondary antibody (1:500 anti-rabbit DL488, Jackson ImmunoLabs, Suffolk, UK) for 3 h at room temperature. After washing, the brains were mounted on slides and images were obtained using a fluorescent microscope equipped with an apotome (Zeiss Axio Imager Z1, Göttingen, Germany). To quantify the effects of rotenone on cell numbers, fly brains of treated and non-treated animals (rotenone treatment for 10 days) were dissected and processed as described above. Z-stack images of rotenone treated and control fly brains were acquired using a. The number of GFP-labeled cells in the central brain (without optic lobes) was determined per brain hemisphere with the Fiji Cell Counter plugin, which facilitates cell counting of 3D images^59^.

### Behavioural testing-Climbing ability (negative geotaxis) assay

Locomotor ability of adult flies was tested by a negative geotaxis assay as described previously^16^ with minor modifications. Twenty male adult flies were placed into a 17 cm long glass tube at a given time point. The flies were tapped to the bottom of the tube and let to climb the tube. After 20 s (or 10 s), a photo was taken in which most of the healthy flies were expected to have crossed the escape line at a height of 6 cm. The height/distance climbed by each fly was analyzed by using Image J.

For the recue experiments, we used the performance index as a quantitative measure^60^. In brief, animals (20 each) were analyzed at the indicated time points by tapping them and counting the number of animals that crossed a 2 cm line within 10 s. The performance index is calculated as follows: PI = 0.5 * ((total number of animals + number of animals above the line − number of animals below the line) divided through the total number of animals). In different types of experiments, we used heights achieved after 10 s or 20 s, depending on preliminary experiments under the respective conditions.

### Tissue collection and analysis

Seventy heads of F1 generation male adult flies from the crossings between *TH-Gal4* and *20xUAS-IVSmCD8::GFP* were dissected in pre-chilled HL3^61^ buffer. Dopaminergic neurons were sorted based on their *mCD8:GFP* expression by magnetic Dynabeads MyOne Streptavidin T1 (Invitrogen, Oslo, Norway) according to a previously described protocol^62^. Briefly, the tissue sample was vortexed for 1 sec, the supernatant was discarded and the procedure was repeated 3–4 times until the supernatant became clear. The heads were transferred to a pre-chilled 7 ml Kontes tissue grinder (Fisher Scientific, Leicestershire, UK), which was rinsed with 1% BSA in HL3 buffer in order to avoid the cells from sticking to the glass surface. About 4 ml of HL3 buffer were added to the tissue grinder and gently using a pestle, the tissues were given 30–32 douncing strokes. The solution was then triturated 5 times using the fire-polished glass pipette narrowed to approximately 50% of the standard tip diameter. The level of dissociation was assessed using a fluorescent microscope with a GFP filter (Axiovert S. 100, Zeiss, Jena, Germany). Then the solution was filtered through a 30 µm cell filter (Miltenyi Biotec, Bergisch Gladbach, Germany). The Dynabeads coupled to undiluted biotinylated rat anti-mouse CD8a antibody (eBioscience, Frankfurt, Germany), was then added to the filtrate and incubated for 1 hour on ice. Following this, the tubes were placed on a MagnaRack (Invitrogen, Karlsuhe, Germany) for 2 min to pellet the beads along with GFP positive cells. The supernatant was removed and discarded. Then the cells were washed three times with ice cold HL3 buffer by putting the tubes on the magnet to remove non-specific cell binding. Cells bound to the coupled beads were resuspended in 30 µl of HL3 buffer and their purity and yield were controlled by taking 5 µl and observe it under the fluorescent microscope. Approximately, 8–10 GFP-positive cells were counted at one time point. Total RNA (from approx. 200 DA neurons) was isolated using the RNA NucleoSpin Tissue Kit (Macherey & Nagel, Düren, Germany). The RNA pellet was resolved in 10 µl RNase-free H_2_O and stored at −80 °C until analyses.

### Microarray analysis

Microarray analyses were performed as described earlier^63,64^. Briefly, cDNA was synthesized by using PrimeScript RT (Takara Bio Europe, Saint Germain-en-Laye, France) according to the manufacturer’s protocol using a CapFinder approach in order to amplify the entire cDNA population^65,66^. The following primers were employed: CapFinderSp6rG (5′-CAG CGG CCG CAG ATT TAG GTG ACA CTA TAG A rGrGrG-3′) and OdT T7 I (5′-GAG AGA GGA TCC AAG TAC TAA TAC GAC TCA CTA TAG GGA GAT TTT TTT TTT TTT TTT TTT T G/A/C-3′). cDNA was amplified with OdT T7 II (5-GAG AGA GGA TCC AAG TAC TAA TAC GAC TCA CTA TAG G-3′) and Adaptor Sp6rG (5′-GAC GCC TGC AGG CGA TGA ATT TAG G-3′) and LA Taq polymerase. *In vitro* transcription of cDNA was performed with MEGAscript® T7 including aminoallyl-UTP and subsequently labeled with Alexa Fluor 647 or 555 (Life Technologies, Darmstadt, Germany) for treated or control sample, respectively. cRNA probes were hybridized to microarray slides (Canadian *Drosophila* Microarray Centre, Toronto, Canada) and scanned with a Gene Pix 4000B scanner equipped with the Gene Pix Pro 6.0 software (Axon Instruments, Science Products, Hofheim, Germany). Data normalization of the probe signal intensity levels across the arrays was performed with Acuity 4.1 (Axon Instruments, Science Products, Hofheim, Germany). A fold change of >1.5 of the mean signal intensity of a specific gene in at least 2 arrays out of three was considered as up-regulated and fold change <0.67 as down-regulated. The enrichment of Gene Ontology terms, and visualization of genes on KEGG (Kyoto Encyclopedia of Genes and Genomes) pathway maps were done with the help of the online FlyBase database (http://flybase.org/), DAVID (Database for Annotation, Visualization and Integrated Discovery^26^ and GOrilla (Gene Ontology enrichment analysis and visualization tool^27^. The microarray raw data have been deposited in the GEO database under the following accession number: GSE74247.

### Quantitative real-time PCR

Quantitative real-time PCR (qRT-PCR) analysis was performed using cDNA samples prepared as described above utilizing a StepOne^TM^ Real-Time PCR system (Applied Biosystems, Darmstadt, Germany) with the DyNAmo Flash SYBR Green qRT-PCR kit (Fisher Scientific, Schwerte, Germany). Ribosomal protein l 32 (*Rpl32)* was used as an internal control gene and expression data were analyzed according to Pfaffl^67^. The primer sets used in this analysis are listed in Table S2.

### Statistical analysis

Statistical analyses were performed using Unpaired-two-tailed Student’s test using Graph Pad Prism software (version 5). The data were presented as mean values +SEM.

## Electronic supplementary material

Supplementary Information
