# Supplementary material for: Impaired Wnt signaling in dopamine containing neurons is associated with pathogenesis in a rotenone triggered Drosophila Parkinson’s disease model
Source: Sci Rep. 2018 Feb 5;8:2372. doi: 10.1038/s41598-018-20836-w (PMC5799194; doi:10.1038/s41598-018-20836-w)
Supplement: Supplementary file 1 — Supplementary Information [file 41598_2018_20836_MOESM1_ESM.docx]

**Supplementary Information**

**Impaired Wnt signaling in dopamine containing neurons is associated with pathogenesis in a rotenone triggered *Drosophila* Parkinson’s disease model**

**Flora Stephano, Stella Nolte, Julia Hoffmann, Samar El-Kholy, Jakob von Frieling, Iris Bruchhaus, Christine Fink, Thomas Roeder**

TABLE S1. TGF-β and Tor signaling pathways-associated genes regulated by rotenone

| **Gene name** | **Symbol** | **Mean** | **SEM** |
| --- | --- | --- | --- |
|  |  |  |  |
| SkpF | skpF | 2.261 | ±0.005 |
| Daughters against dpp | Dad | 1.842 | ±0.100 |
| Smad anchor for receptor activation | Sara | 1.364 | ±0.095 |
| Rho-kinase A | Rok | 1.335 | ±0.060 |
| Eukaryotic initiation factor 4B | elF-4B | 1.870 | ±0.185 |
| Akt1 | Akt1 | 1.409 | ±0.110 |
| Target of rapamycin | Tor | 2.228 | ±0.016 |

TABLE S2. Oligonucleotides used for qRT-PCR analysis

| Name | Sequence (5’ to 3’) |
| --- | --- |
| Rpl32-sense | TTGGCTTCGGTTTCCGGCAAG |
| Rpl32-antisense | ATCGATCCGACTGGTGGCGGAT |
| Dad-sense | CAGATCCACTCGGTGGGTGCC |
| Dad-antisense | CAGATCCACTCGGTGGTGCC |
| Armadillo-sense | CTCATTCGCCAGCAGTCGGT |
| Armadillo-antisense | CAAAGAACGCCAGCAGCCAC |
| Spitz-sense | AATATTGGGCCTGGGCGTGG |
| Spitz-antisense | CCGCGCCTCTTCGATCTCCTC |
| l(2)NC136-sense | TCCTACTGTGGCAGCTATCG |
| l(2)NC136-antisense | ACTGTCCGAGACAGGAAGAC |
| CaM-sense | GCTGCAGGACATGATCAACG |
| CaM-antisense | TCTCGGATCTCCTCTTCGCT |
| Dat-sense | GGCGCATTAGGCCCACTTAT |
| Dat-antisense | GCTTCCCAGAGACGGTCAAT |


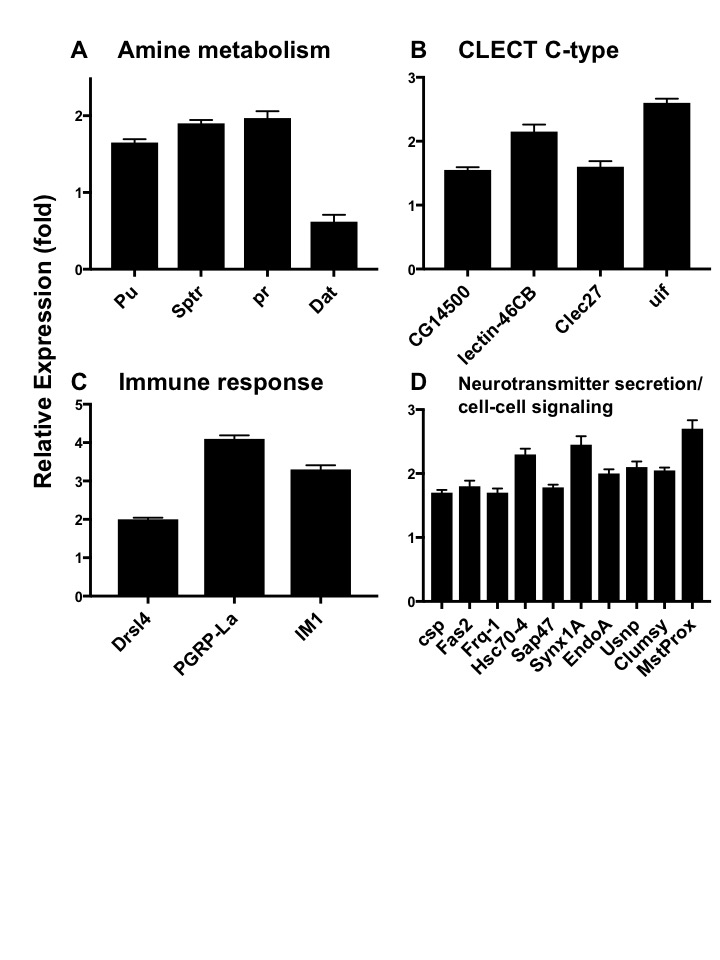


*FIG. S1. Gene expression pattern changes in cellular processes affected by rotenone. Functional categories overrepresented in the lists of genes differentially expressed in rotenone treated flies identified by DAVID (*[*http://david.abcc.ncifcrf.gov/*](http://david.abcc.ncifcrf.gov/)*), (Table 1, p < 0.05). Fold changes for genes in regulation of (A) Amine metabolism, (B) c- type lectins and (C) Immune response (D) Neurotransmitter secretion/cell-cell signaling. Error bars represent SEM. Pu: punch, pr: purple, Sptr:, Sepiapterin reductase, Dat: Dopamine N acetyltransferase, lectin-46Cb, CG14500, Clect27: C-type lectin 27kD, uif: Uninflatable, PGRP-LA: Peptidoglycan recognition protein LA, Drsl4: Drosomycin like 4, Im1: Immune induced molecule 1.*

Fold transcription

*

**

***

*FIG. S2. Verification of differential expression using qRT-PCR. For three selected genes, the reduced expression (Dat - the Dopamine N-acetyltransferase) or the increased expression (calmodulin – cam; l(2)Nc136: lethal2NC136) was confirmed by qRT-PCR. N = 3, mean ± SEM.*
